# Chinese Herbal Medicine Reduces the Risk of Heart Failure in Hypertensive Patients: A Nationwide, Retrospective, Cohort Study

**DOI:** 10.3389/fcvm.2022.922728

**Published:** 2022-07-11

**Authors:** Chun-Ting Liu, I-Ling Hung, Chung Y. Hsu, Kai-Chieh Hu, Yung-Hsiang Chen, Ming-Yen Tsai

**Affiliations:** ^1^Department of Chinese Medicine, Kaohsiung Chang Gung Memorial Hospital, Chang Gung University College of Medicine, Kaohsiung, Taiwan; ^2^Graduate Institute of Integrated Medicine, College of Chinese Medicine, China Medical University, Taichung, Taiwan; ^3^Department of Chinese Medicine, Dali Branch, Jen-Ai Hospital, Taichung, Taiwan; ^4^Graduate Institute of Biomedical Sciences, China Medical University, Taichung, Taiwan; ^5^Management Office for Health Data, College of Medicine, China Medical University Hospital, Taichung, Taiwan; ^6^Department of Psychology, College of Medical and Health Science, Asia University, Taichung, Taiwan

**Keywords:** hypertension, heart failure, pharmaco-epidemiology, National Health Insurance Research Database, Chinese herbal medicine

## Abstract

**Background:**

Hypertension (HTN) is the leading preventable risk factor for cardiovascular disease worldwide. Patients with HTN are at higher risk for heart failure (HF). The currently available therapeutic approaches for HTN do not always optimally control blood pressure or are not suitable for hypertensive patients who have a higher number of comorbidities. This study aimed to determine whether Chinese herbal medicine (CMH)-based interventions could reduce the risk of HF in hypertensive patients.

**Methods:**

This retrospective study randomly selected 2 million enrollees from the National Health Insurance Research Database and identified 507,608 patients who were newly diagnosed with HTN in 2000–2017. After 1:1 frequency-matching by age, sex, index year, income, urbanization, duration of HTN, comorbidities and antihypertensive medications, we selected 8,912 eligible patients in each group. During 16 years of follow-up, 380 CHM users and 426 CHM non-users developed HF, representing incidence rates of 6.29 and 7.43 per 1,000 person-years, respectively.

**Results:**

CHM users had significantly lower HF risk compared with CHM non-users (adjusted HR = 0.85, 95% CI 0.74–0.98). The markedly predominant effect was observed in those receiving CHM products for more than 180 days (adjusted HR = 0.65). The frequently prescribed formula, Jia-Wei-Xiao-Yao-San, and the single herbs Ge Gen, Huang Qi, Du Zhong, Huang Qin, and Chuan Xiong were significantly associated with lower risk of HF.

**Conclusions:**

This population-based study revealed decreased HF risk in hypertensive patients with CHM use. These findings may provide a reference for HF prevention strategies and support the integration of CHM into clinical intervention programs that provide a favorable prognosis for hypertensive patients.

## Introduction

Hypertension (HTN) is the leading preventable risk factor for cardiovascular disease (CVD) and premature death worldwide (1, 2). The prevalence of HTN is rising globally, especially in low- and middle-income countries (3). In 2010, an estimated 1.39 billion people, equaling 31.1% of the global adult population, had HTN, defined as systolic blood pressure (BP) ≥ 140 mm Hg, diastolic BP ≥ 90 mm Hg, and/or current use of antihypertensive medication (3). The prevalence of HTN was higher in men than in women and was lower in high-income countries than in low and middle-income countries (3). Rural residents had a higher prevalence of HTN than urban residents in high and middle-income countries, but the trend was reversed in low-income countries (3). These differences in prevalence are not fully understood but are likely associated with differences in disease awareness, unhealthy diet, physical activity and healthcare resources at different urbanization levels and socioeconomic statuses (3). Heart failure (HF) is also an increasing global pandemic, affecting at least 64.3 million people worldwide (4). HTN is important risk factor for HF and is comorbid in most patients with HF (4, 5). A previous study showed that 20 mmHg elevation of systolic BP is associated with 50% increased risk of HF (6). In the Framingham Heart Study cohort, 91% of the participants developed HTN before HF, and compared with normotensive individuals, the male and female patients with HTN had 2- and 3-fold increased risks of HF, respectively (7). Elevated BP changes the structure and function of blood vessels and the left ventricle (LV), and prolonged exposure to an increasing load can lead to LV structural remodeling (8). Myocardial hypertrophy is a compensatory mechanism in response to the remodeled LV, and HF can develop as a consequence of increased LV stiffness and the presence of diastolic dysfunction (9).

Intervention to prevent or attenuate BP rise could lead to substantial reductions in HF development and further morbidity and mortality risks (10). Despite the effectiveness of lifestyle modifications and widespread use of antihypertensive medications, the proportions of HTN awareness, treatment and lifestyle modification are low (11). Therefore, HTN continues to be an important public health challenge worldwide.

Chinese herbal medicine (CHM) has been used for thousands of years to treat or prevent diseases, including HTN. Two systemic reviews recently reported that CHM appeared to have significant antihypertensive effects and may possibly improve outcomes in CVD and HF (12, 13). At present, most population-based studies have focused on the potential antihypertensive effects and prescription patterns of CHM in patients with HTN (14–16). To the best of our knowledge, no studies have investigated the use of CHM for reducing the risk of HF in hypertensive patients. The purpose of this study was to investigate whether the use of CHM can reduce the risk of HF in hypertensive patients. The hypothesis of the study was that the risk of HF would be further reduced by CHM in patients with HTN.

## Methods

### Data Source

In Taiwan, de-identified personal medical claims from the National Health Insurance Research Database (NHIRD), derived from the National Health Insurance (NHI) program, are available for research purposes. Information in the database contains gender, birth date, details of inpatient and outpatient orders, diagnostic codes from the International Classification of Diseases, 9th and 10th edition, Clinical Modification (ICD-9-CM and ICD-10-CM), and medicines prescribed. This study used a longitudinal cohort population-based database, which consists of 2 million randomly selected beneficiaries from the NHIRD. The study was approved by the Research Ethics Committee at the China Medical University and Hospital in Taiwan (CMUH109-REC2-031).

### Study Population

**Figure 1:**
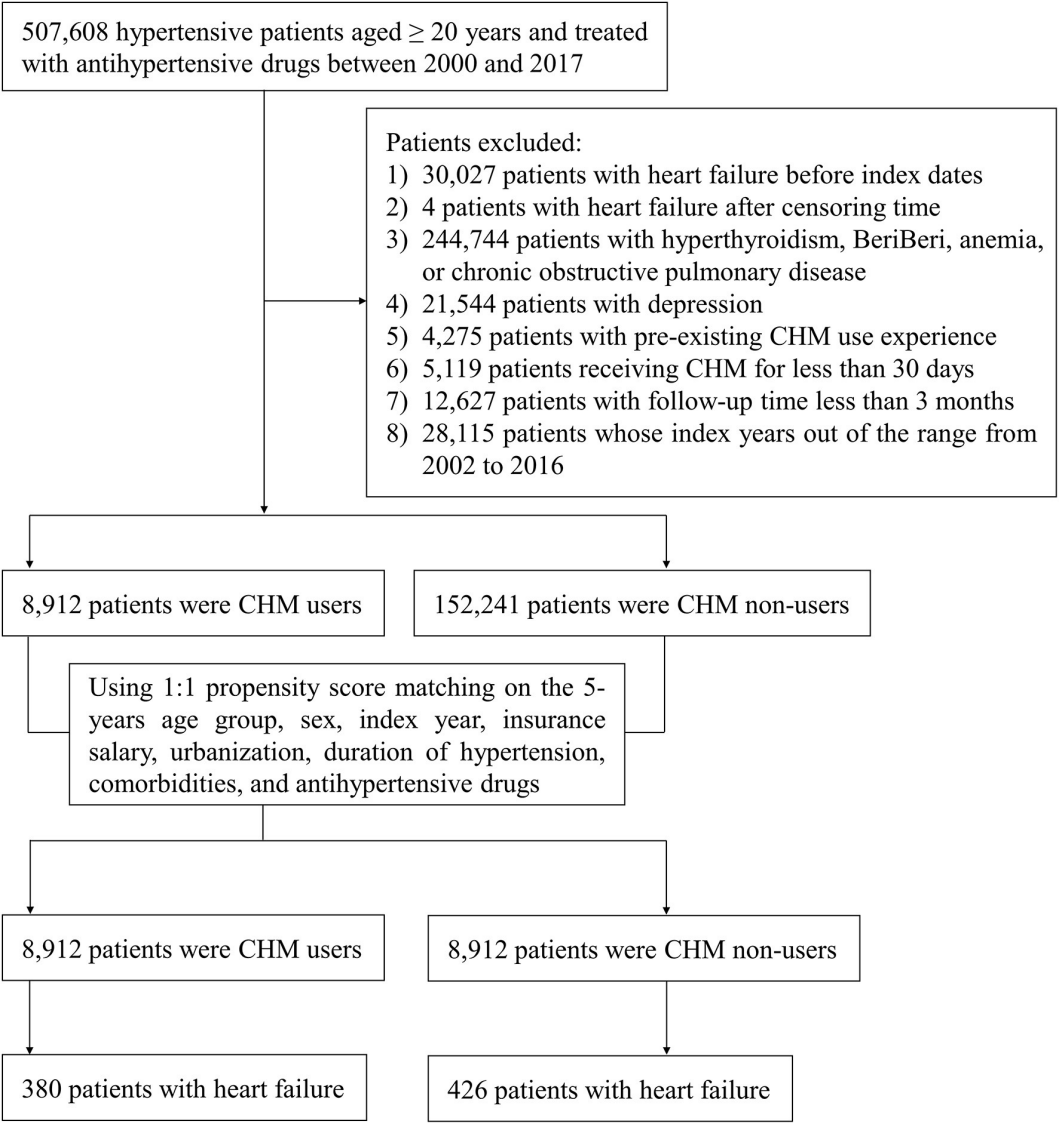
Flowchart of the study participant selection.

A total of 507,608 hypertensive (ICD-9-CM: 401-405; ICD-10-CM: I10-I15) patients aged ≥ 20 years and treated with antihypertensive drugs for at least 30 days between 2000 and 2017 were identified. Antihypertensive drugs included diuretics, α-blockers, β-blockers, angiotensin receptor blockers (ARB), calcium channel blockers (CCB), angiotensin-converting enzyme inhibitors (ACEI), and others, i.e., hydralazine, clonidine, nitroprusside, methyldopa, minoxidil, and diazoxide. All hypertensive patients had at least two outpatient visits or one hospital admission. The primary outcome was HF (ICD-9-CM: 428; ICD-10-CM: I50). The ICD-9 diagnostic codes used in the study have been shown in previous validation studies to have high positive predictive values of 88.5 and 97.6% for HTN and HF, respectively (17). To improve the accuracy of diagnosis of HF, patients with HF should be admitted to hospital at least once or visit outpatient clinics at least three times within a year. Each patient was either followed up from the index date until the onset of HF or until the patient was censored (e.g., death, withdrawal from the NHI program, or 31st December 2017). A total of 30,031 patients who had HF before the initial diagnosis of HTN or were outside the range of the censoring time were excluded. Among 244,744 patients diagnosed with hyperthyroidism (ICD-9-CM: 242; ICD-10-CM: E05), BeriBeri (ICD-9-CM: 265.0; ICD-10-CM: E51.11, E51.12), anemia (ICD-9-CM: 280-285; ICD-10-CM: D46.1, D46.4, D50-D59, D60-D64), or chronic obstructive pulmonary disease (ICD-9-CM: 490-496; ICD-10-CM: J40-J47, J67) and 21,544 patients diagnosed with depression (ICD-9-CM: 296.2, 296.3, 296.82, 298.0, 311; ICD-10-CM: F32, F33) were excluded because these diseases may be associated with higher BP values. CHM use was determined from provider prescription records available from the NHIRD. Among the hypertensive patients, those who had received CHM for at least 30 days were defined as CHM users, and those who had not received CHM were defined as CHM non-users. Also excluded were 4,275 patients with pre-existing CHM use and 5,119 patients who had received CHM for <30 days due to the potential confounding effects. A total of 12,627 patients with follow-up times of <3 months following antihypertensive drug therapy were excluded because they were not regarded as having received adequate treatment. The index dates for CHM users were the first dates when the hypertensive patients started taking CHM for HTN; the index dates for CHM non-users were dates randomly selected from the range of antihypertensive drug prescription dates to the censoring time. Because the database began in 2000, the index years to explore comorbidities and concomitant medications started in 2002. Among 28,115 patients whose index years were outside the range of 2002 to 2016 were excluded as well. Pre-existing medical conditions, coronary heart disease (CHD) (ICD-9-CM: 410-414, 429; ICD-10-CM: I20-I25), cerebrovascular disease (CVD) (ICD-9-CM: 430-438; ICD-10-CM: I60-I69), diabetes mellitus (DM) (ICD-9-CM: 250; ICD-10-CM: E08-E13), hyperlipidemia (ICD-9-CM: 272.0-272.4; ICD-10-CM: E78.0-E78.5), and peripheral arterial occlusive disease (ICD-9-CM: 440; ICD-10-CM: I70, I75) were considered as confounding factors. At least two outpatient records or at least one inpatient record for the diseases were regarded as comorbidities. CHM users and non-users were matched for 5-year age group, sex, index year, income, urbanization, duration of HTN, comorbidities and antihypertensive medications by the propensity score in a 1:1 ratio. In total, 8,912 CHM users and 8,912 CHM non-users were included in the study. A flowchart illustrating the study participant selection is shown in Figure 1.

### Statistical Analysis

Statistical analyses were performed in SAS 9.4 software (SAS Institute Inc., Cary, NC). Standardized mean differences (SMDs) were used to test for homogeneity of distributions in baseline characteristics, comorbidities, and medications between CHM users and non-users. The incidence rate (IR) of HF was expressed as the number of HF per 1,000 person-years of follow-up. The cumulative incidence of HF was estimated by the Kaplan–Meier method. The difference between cumulative incidence curves for CHM users and non-users was examined with the log-rank test. The relative risks of HF were expressed as hazard ratios (HRs) with 95% confidence intervals (95% CIs) and were estimated by univariate Cox proportional-hazards models. Adjusted hazard ratios (aHRs) with 95% CIs were estimated by multivariate Cox proportional-hazards models with covariates of gender, age, income, urbanization, duration of HTN, comorbidities and antihypertensive medications. A *p*-value of < 0.05 was considered statistically significant.

## Results

### Demographic Characteristics of Study Populations

A total of 8,912 eligible patients in each group were included in the analysis. Table 1 shows baseline characteristics, comorbidities, and medications of hypertensive patients with and without CHM treatment. CHM users tended to be male and middle-aged (45–65 years old). There was no difference in duration of HTN between the two groups (SMD = 0.0065, which is <0.1). After propensity score matching, there were no significant differences in gender, age, income, urbanization, comorbidities, or antihypertensive drugs used between CHM users and non-users. Therefore, the two groups had similar conditions in terms of sex, age, income, urbanization, comorbidities, antihypertensive medications, duration of HTN, and follow-up time for the following analyses.

**Table 1:** Baseline characteristics, comorbidities, and medications of hypertensive patients with and without CHM treatment.

**Variable**	**CHM**		**SMD^§^**
	**No**	**Yes**	
	***n*** **(%)/mean ±SD**	***n*** **(%)/mean ±SD**	
**All**	8,912 (50.00)	8,912 (50.00)	
**Sex**			
Female	4,191 (47.03)	4,147 (46.53)	0.0099
**Age (year)**			
<45	2,787 (31.27)	2,769 (31.07)	0.0044
45–65	5,066 (56.84)	5,183 (58.16)	0.0266
>65	1,059 (11.88)	960 (10.77)	0.0351
sMean ± SD	50.53 ± 11.91	50.33 ± 11.56	0.0351
**Income (NTD/month)**			
≤ 17,880	1,755 (19.69)	1,721 (19.31)	0.0096
17,881–43,900	5,136 (57.63)	5,192 (58.26)	0.0127
≥43,901	2,021 (22.68)	1,999 (22.43)	0.0059
**Urbanization**			
Urban	4,907 (55.06)	4,861 (54.54)	0.0104
Suburban	3,226 (36.20)	3,305 (37.08)	0.0184
Rural	779 (8.74)	746 (8.37)	0.0132
**Comorbidities**			
Coronary heart disease	2,602 (29.20)	2,561 (28.74)	0.0101
Cerebrovascular disease	1,506 (16.90)	1,423 (15.97)	0.0251
Diabetes mellitus	2,218 (24.89)	2,250 (25.25)	0.0083
Hyperlipidemia	4,352 (48.83)	4,363 (48.96)	0.0025
PAOD	212 (2.38)	210 (2.36)	0.0015
**Medications**			
ACEI	4,515 (50.66)	4,490 (50.38)	0.0056
ARB	6,801 (76.31)	6,818 (76.50)	0.0045
α-blockers	2,704 (30.34)	2,695 (30.24)	0.0022
β-blockers	6,663 (74.76)	6,654 (74.66)	0.0023
CCB	8,029 (90.09)	8,030 (90.10)	0.0004
Diuretics	4,513 (50.64)	4,539 (50.93)	0.0058
Others	972 (10.91)	990 (11.11)	0.0065
**Duration of hypertension at index date (year)**	5.35 ± 3.98	5.38 ± 4.15	0.0065
**Follow-up time (year)**	6.43 ± 4.02	6.78 ± 3.89	0.0874

§ *A SMD of ≤ 0.1 indicates a negligible difference between the two cohorts*.

*ACEI, Angiotensin-Converting Enzyme Inhibitors; ARB, Angiotensin Receptor Blockers; CCB, Calcium Channel Blockers; CHM, Chinese Herbal Medicine; NTD, New Taiwan Dollar; PAOD, Peripheral Arterial Occlusive Disease; SD, Standard Deviation; SMD, Standardized Mean Difference*.

### The Incidence Rate and Hazard Ratio of Heart Failure in Hypertensive Patients

**Figure 2:**
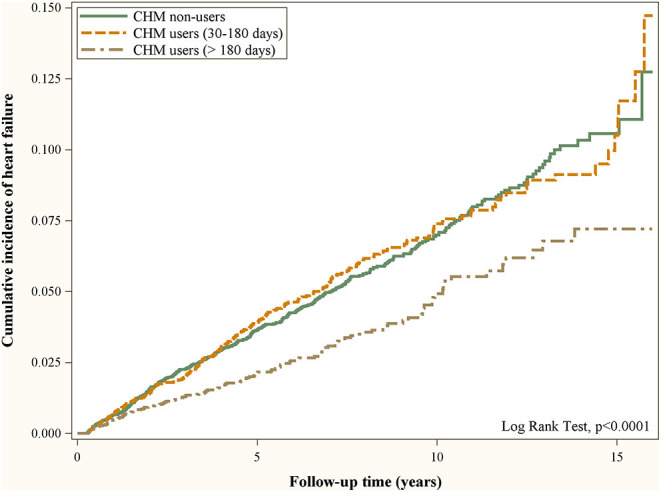
Cumulative incidence of heart failure in hypertensive patients with and without CHM treatment using the Kaplan–Meier method.

During 16 years of follow-up, 380 CHM users and 426 CHM non-users developed HF. Table 2 lists the IRs and the crude and adjusted HRs with 95% CIs of HF between hypertensive patients with and without CHM treatment. The IR of HF in CHM users (6.29 per 1,000 person-years) was lower than that in CHM non-users (7.43 per 1,000 person-years), and it was found that CHM was associated with a significantly decreased risk of HF [aHR = 0.85, 95% CI = (0.74, 0.98), *p* = 0.0264]. In addition, a higher number of days of receiving medicine was associated with less risk of HF [>180 days: aHR = 0.65, 95% CI = (0.53, 0.79), *p* < 0.0001]. These findings indicated that adjuvant CHM treatment for more than 180 days reduced the HF risk of hypertensive patients. Figure 2 presents the cumulative incidence of HF in hypertensive patients with and without CHM treatment using the Kaplan–Meier method. The figure indicates that hypertensive patients with CHM treatment had a significantly lower probability of HF than their counterparts without CHM treatment did, and the probability of HF events was the lowest among hypertensive patients who received CHM for more than 180 days (log-rank *p*-value <0.0001).

**Table 2:** IRs and crude and adjusted HRs with 95% CIs of heart failure between hypertensive patients with and without CHM treatment.

**Variable**	**Event**	**Person-years**	**IR**	**Crude**		Adjusted^*^	
	***n*** **= 806**		**1,000 person-years**	**HR (95% CI)**	***P*** **-value**	**HR (95% CI)**	***P*** **-value**
**CHM non-users**	426	57,336	7.43	1 (Reference)		1 (Reference)	
**CHM users**	380	60,419	6.29	0.85 (0.74, 0.97)	0.0172	0.85 (0.74, 0.98)	0.0264
30–180 days	255	33,539	7.60	1.02 (0.87, 1.19)	0.8041	1.02 (0.87, 1.19)	0.8391
>180 days	125	26,881	4.65	0.63 (0.51, 0.76)	<0.0001	0.65 (0.53, 0.79)	<0.0001

* *Adjusted for gender, age, income, urbanization, duration of hypertension, coronary heart disease, cerebrovascular disease, diabetes mellitus, hyperlipidemia, PAOD, ACEI, ARB, α-blockers, β-blockers, CCB, diuretics, and others, i.e., hydralazine, clonidine, nitroprusside, methyldopa, minoxidil, and diazoxide*.

*ACEI, Angiotensin-Converting Enzyme Inhibitors; ARB, Angiotensin Receptor Blockers; CCB, Calcium Channel Blockers; CHM, Chinese Herbal Medicine; CI, Confidence Interval; HR, Hazard Ratio; IR, Incidence Rate; PAOD, Peripheral Arterial Occlusive Disease*.

Table 3 shows Cox regression analyses of HF associated with covariates among hypertensive patients. Men were at higher risk of HF than women were [aHR = 1.34, 95% CI = (1.15, 1.57), *p* = 0.0002], and HF was increasingly common with advancing age [45–65 years: aHR = 1.63, 95% CI = (1.31, 2.02), *p* < 0.0001; > 65 years: aHR = 5.30, 95% CI = (4.17, 6.72), *p* < 0.0001]. Levels of income or urbanization were not associated with the development of HF in hypertensive patients. Hypertensive patients with CHD, CVD or diabetes mellitus had a higher risk of HF [CHD: aHR = 1.55, 95% CI = (1.34, 1.80), *p* < 0.0001; CVD: aHR = 1.30, 95% CI = (1.11, 1.53), *p* = 0.0014; diabetes mellitus: aHR = 1.75, 95% CI = (1.50, 2.04), *p* < 0.0001]. Use of ACEI or diuretics increased the risk of HF [ACEI: aHR = 1.45, 95% CI = (1.23, 1.69), *p* < 0.0001; diuretics: aHR = 1.68, 95% CI = (1.42, 1.99), *p* < 0.0001]. However, use of ARB reduced the risk of HF [ARB: aHR = 0.57, 95% CI = (0.49, 0.67), *p* < 0.0001]. These findings indicated that hypertensive patients who were male or had concomitant CHD, CVD, or diabetes mellitus were at a higher risk of HF.

**Table 3:** Cox regression analyses of heart failure associated with covariates among hypertensive patients.

**Variable**	**Event**	**Person-years**	**IR**	**Crude**		Adjusted^*^	
	***n*** **= 806**		**1,000 person-years**	**HR (95% CI)**	***P*** **-value**	**HR (95% CI)**	***P*** **-value**
**Sex**							
Female	396	56,299	7.03	1 (Reference)		1 (Reference)	
Male	410	61,456	6.67	0.95 (0.83, 1.09)	0.4603	1.34 (1.15, 1.57)	0.0002
**Age (year)**							
<45	113	37,232	3.03	1 (Reference)		1 (Reference)	
45–65	412	68,515	6.01	1.98 (1.61, 2.44)	<0.0001	1.63 (1.31, 2.02)	<0.0001
>65	281	12,008	23.40	7.76 (6.24, 9.65)	<0.0001	5.30 (4.17, 6.72)	<0.0001
**Income (NTD/month)**							
≤ 17,880	206	23,098	8.92	1 (Reference)		1 (Reference)	
17,881–43,900	479	68,413	7.00	0.79 (0.67, 0.93)	0.0040	1.05 (0.89, 1.24)	0.5397
≥43,901	121	26,243	4.61	0.52 (0.41, 0.65)	<0.0001	0.84 (0.67, 1.06)	0.1428
**Urbanization**							
Urban	442	64,785	6.82	1 (Reference)		1 (Reference)	
Suburban	284	43,100	6.59	0.97 (0.83, 1.12)	0.6558	0.88 (0.75, 1.02)	0.0865
Rural	80	9,870	8.11	1.19 (0.94, 1.51)	0.1537	0.96 (0.75, 1.22)	0.7358
**Comorbidities**							
Coronary heart disease							
No	428	85,064	5.03	1 (Reference)		1 (Reference)	
Yes	378	32,691	11.56	2.31 (2.01, 2.65)	<0.0001	1.55 (1.34, 1.80)	<0.0001
Cerebrovascular disease							
No	579	100,300	5.77	1 (Reference)		1 (Reference)	
Yes	227	17,455	13.00	2.27 (1.94, 2.64)	<0.0001	1.30 (1.11, 1.53)	0.0014
Diabetes mellitus							
No	486	91,238	5.33	1 (Reference)		1 (Reference)	
Yes	320	26,517	12.07	2.29 (1.99, 2.64)	<0.0001	1.75 (1.50, 2.04)	<0.0001
Hyperlipidemia							
No	393	65,038	6.04	1 (Reference)		1 (Reference)	
Yes	413	52,718	7.83	1.31 (1.14, 1.51)	0.0001	1.05 (0.91, 1.22)	0.5168
PAOD							
No	779	115,146	6.77	1 (Reference)		1 (Reference)	
Yes	27	2,609	10.35	1.53 (1.04, 2.25)	0.0296	0.91 (0.62, 1.34)	0.6360
**Medications**							
ACEI							
No	246	52,536	4.68	1 (Reference)		1 (Reference)	
Yes	560	65,220	8.59	1.83 (1.57, 2.13)	<0.0001	1.45 (1.23, 1.69)	<0.0001
ARB							
No	227	25,432	8.93	1 (Reference)		1 (Reference)	
Yes	579	92,324	6.27	0.70 (0.60, 0.81)	<0.0001	0.57 (0.49, 0.67)	<0.0001
α-blockers							
No	471	78,589	5.99	1 (Reference)		1 (Reference)	
Yes	335	39,167	8.55	1.42 (1.24, 1.64)	<0.0001	0.90 (0.77, 1.05)	0.1884
β-blockers							
No	141	24,690	5.71	1 (Reference)		1 (Reference)	
Yes	665	93,065	7.15	1.24 (1.03, 1.49)	0.0200	1.06 (0.88, 1.29)	0.5388
CCB							
No	55	9,767	5.63	1 (Reference)		1 (Reference)	
Yes	751	107,988	6.95	1.23 (0.93, 1.61)	0.1450	0.90 (0.67, 1.19)	0.4435
Diuretics							
No	218	52,832	4.13	1 (Reference)		1 (Reference)	
Yes	588	64,923	9.06	2.19 (1.88, 2.56)	<0.0001	1.68 (1.42, 1.99)	<0.0001
Others							
No	683	102,996	6.63	1 (Reference)		1 (Reference)	
Yes	123	14,759	8.33	1.25 (1.03, 1.52)	0.0224	0.93 (0.76, 1.13)	0.4573

* *Adjusted for gender, age, income, urbanization, duration of hypertension, coronary heart disease, cerebrovascular disease, diabetes mellitus, hyperlipidemia, PAOD, ACEI, ARB, α-blockers, β-blockers, CCB, diuretics, and others, i.e. hydralazine, clonidine, nitroprusside, methyldopa, minoxidil, and diazoxide*.

*ACEI, Angiotensin-Converting Enzyme Inhibitors; ARB, Angiotensin Receptor Blockers; CCB, Calcium Channel Blockers; CHM, Chinese Herbal Medicine; CI, Confidence Interval; HR, Hazard Ratio; IR, Incidence Rate; NTD, New Taiwan Dollar; PAOD, Peripheral Arterial Occlusive Disease*.

Table 4 presents the IRs and the crude and adjusted HRs with 95% CIs of HF between hypertensive patients with and without CHM treatment, stratified by baseline characteristics, comorbidities, and medications. Among women, CHM users were at a lower risk of HF [aHR = 0.75, 95% CI = (0.61, 0.91), *p* = 0.0046], and CHM was associated with a significantly decreased risk of HF in older women [45–65 years: aHR = 0.75, 95% CI = (0.56, 0.99), *p* = 0.0431; >65 years: aHR = 0.71, 95% CI = (0.52, 0.96), *p* = 0.0278]. Among men, CHM was not associated with a significantly decreased risk of HF. For higher-income earners, CHM users were more likely to reduce the risk of HF [aHR = 0.63, 95% CI = (0.43, 0.91), *p* = 0.0131]. For rural residents, CHM users were more likely to reduce the risk of HF [aHR = 0.59, 95% CI = (0.37, 0.93), *p* = 0.0231]. CHM did not reduce the risk of HF for hypertensive patients with comorbidities such as CHD, CVD, DM, hyperlipidemia, or peripheral arterial occlusive disease. Among antihypertensive drugs, only β-blockers along with CHM could significantly reduce the risk of HF [aHR = 0.84, 95% CI = (0.72, 0.98), *p* = 0.0273].

**Table 4:** IRs and crude and adjusted HRs with 95% CIs of heart failure between hypertensive patients with and without CHM treatment, stratified by baseline characteristics, comorbidities, and medications.

**Variable**	**CHM non-users**			**CHM users**			**Crude**		Adjusted^*^	
	**Event**	**Person-year**	**IR**	**Event**	**Person-year**	**IR**	**HR (95% CI)**	***P*** **-value**	**HR (95% CI)**	***P*** **-value**
**Female**	227	27,652	8.21	169	28,647	5.90	0.72 (0.59, 0.87)	0.0010	0.75 (0.61, 0.91)	0.0046
Aged <45 years	10	6,229	1.61	14	6,722	2.08	1.27 (0.56, 2.86)	0.5662	1.22 (0.54, 2.78)	0.6327
Aged 45–65 years	109	17,652	6.17	85	18,227	4.66	0.75 (0.56, 0.99)	0.0445	0.75 (0.56, 0.99)	0.0431
Aged > 65 years	108	3,770	28.64	70	3,698	18.93	0.66 (0.49, 0.89)	0.0073	0.71 (0.52, 0.96)	0.0278
**Male**	199	29,684	6.70	211	31,772	6.64	0.99 (0.82, 1.20)	0.9389	0.98 (0.81, 1.19)	0.8319
Aged <45 years	48	11,981	4.01	41	12,301	3.33	0.84 (0.55, 1.27)	0.4114	0.84 (0.55, 1.27)	0.4059
Aged 45–65 years	105	15,461	6.79	113	17,174	6.58	0.97 (0.74, 1.26)	0.8176	0.98 (0.75, 1.28)	0.8623
Aged > 65 years	46	2,242	20.52	57	2,297	24.81	1.22 (0.83, 1.80)	0.3176	1.27 (0.86, 1.88)	0.2334
**Income (NTD/month)**										
≤ 17,880	108	11,287	9.57	98	11,812	8.30	0.87 (0.66, 1.14)	0.2993	0.83 (0.63, 1.09)	0.1848
17,881–43,900	244	33,217	7.35	235	35,197	6.68	0.91 (0.76, 1.09)	0.2890	0.92 (0.77, 1.10)	0.3579
≥43,901	74	12,832	5.77	47	13,411	3.50	0.61 (0.42, 0.88)	0.0075	0.63 (0.43, 0.91)	0.0131
**Urbanization**										
Urban	224	31,876	7.03	218	32,909	6.62	0.94 (0.78, 1.14)	0.5373	0.95 (0.78, 1.14)	0.5604
Suburban	152	20,681	7.35	132	22,419	5.89	0.80 (0.63, 1.01)	0.0577	0.82 (0.65, 1.04)	0.0965
Rural	50	4,779	10.46	30	5,092	5.89	0.56 (0.36, 0.89)	0.0130	0.59 (0.37, 0.93)	0.0231
**Comorbidities**										
Coronary heart disease	204	16,219	12.58	174	16,472	10.56	0.84 (0.68, 1.03)	0.0865	0.87 (0.71, 1.06)	0.1750
Cerebrovascular disease	127	8,531	14.89	100	8,925	11.20	0.75 (0.58, 0.98)	0.0356	0.83 (0.64, 1.08)	0.1680
Diabetes mellitus	169	12,672	13.34	151	13,846	10.91	0.81 (0.65, 1.01)	0.0668	0.82 (0.66, 1.03)	0.0820
Hyperlipidemia	217	25,814	8.41	196	26,904	7.29	0.87 (0.72, 1.05)	0.1521	0.87 (0.72, 1.06)	0.1657
PAOD	14	1,295	10.81	13	1,314	9.89	0.92 (0.43, 1.96)	0.8320	0.71 (0.31, 1.62)	0.4142
**Medications**										
ACEI	288	31,904	9.03	272	33,316	8.16	0.90 (0.76, 1.06)	0.2179	0.90 (0.76, 1.06)	0.2105
ARB	301	45,217	6.66	278	47,107	5.90	0.89 (0.75, 1.04)	0.1430	0.87 (0.74, 1.03)	0.1070
α-blocker	163	19,146	8.51	172	20,021	8.59	1.01 (0.82, 1.25)	0.9251	0.97 (0.78, 1.20)	0.7634
β-blocker	353	45,346	7.78	312	47,720	6.54	0.84 (0.72, 0.98)	0.0241	0.84 (0.72, 0.98)	0.0273
CCB	392	52,665	7.44	359	55,324	6.49	0.87 (0.75, 1.00)	0.0580	0.88 (0.76, 1.01)	0.0745
Diuretics	308	31,501	9.78	280	33,422	8.38	0.86 (0.73, 1.01)	0.0599	0.87 (0.74, 1.02)	0.0918
Others	58	7,095	8.17	65	7,664	8.48	1.02 (0.72, 1.46)	0.9031	1.03 (0.72, 1.47)	0.8821

* *Adjusted for gender, age, income, urbanization, duration of hypertension, coronary heart disease, cerebrovascular disease, diabetes mellitus, hyperlipidemia, PAOD, ACEI, ARB, α-blockers, β-blockers, CCB, diuretics, and others, i.e., hydralazine, clonidine, nitroprusside, methyldopa, minoxidil, and diazoxide*.

*ACEI, Angiotensin-Converting Enzyme Inhibitors; ARB, Angiotensin Receptor Blockers; CCB, Calcium Channel Blockers; CHM, Chinese Herbal Medicine; CI, Confidence Interval; HR, Hazard Ratio; IR, Incidence Rate; NTD, New Taiwan Dollar; PAOD, Peripheral Arterial Occlusive Disease*.

### Ten Most Common Used Single- and Multi-Herb CHM Drugs in Hypertensive Patients

Table 5 presents the crude and adjusted HRs with 95% CIs of HF associated with the ten most commonly used single- and multi-CHM drugs in hypertensive patients. The most common CHM formula prescribed was Tian-Ma-Gou-Teng-Yin (TMGTY), and the most common single herb prescribed was Dan Shen. As compared to hypertensive patients not receiving CHM treatment, those who received Ge Gen [aHR = 0.61, 95% CI = (0.44, 0.86), *p* = 0.0046], Huang Qi [aHR = 0.51, 95% CI = (0.32, 0.81), *p* = 0.0043], Du Zhong [aHR = 0.69, 95% CI = (0.48, 0.99), *p* = 0.0443], Huang Qin [aHR = 0.56, 95% CI = (0.37, 0.86), *p* = 0.0076], Chuan Xiong [aHR = 0.37, 95% CI = (0.22, 0.65), *p* = 0.0005], or Jia-Wei-Xiao-Yao-San (JWXYS) [aHR = 0.60, 95% CI = (0.41, 0.88), *p* = 0.0086] had a lower risk of HF.

**Table 5:** Crude and adjusted HRs with 95% CIs of heart failure associated with ten most common used single- and multi-herb CHM drugs in hypertensive patients.

**Variable**	**Frequency**	**Average duration** **(days)**	**Daily dose** **(g)**	**Crude**		Adjusted^*^	
				**HR (95% CI)**	***P*** **-value**	**HR (95% CI)**	***P*** **-value**
**Single-herb products**							
Dan Shen (*Rx. Salviae Miltiorrhizae*)	11,234	11.69	1.67	0.85 (0.67, 1.09)	0.1964	0.87 (0.68, 1.11)	0.2651
Gou Teng (*Ram. cum Uncis Uncariae*)	7,576	11.19	1.80	0.70 (0.52, 0.93)	0.0133	0.75 (0.57, 1.00)	0.0536
Da Huang (*Rx. et Rz. Rhei*)	5,749	11.12	0.64	1.08 (0.79, 1.49)	0.6235	1.06 (0.77, 1.46)	0.7252
Ge Gen (*Rx. Puerariae*)	5,417	10.48	1.87	0.55 (0.39, 0.77)	0.0005	0.61 (0.44, 0.86)	0.0046
Huang Qi (*Rx. Astragali*)	4,048	11.89	1.69	0.52 (0.33, 0.83)	0.0060	0.51 (0.32, 0.81)	0.0043
Du Zhong (*Cx. Eucommiae*)	3,920	11.30	1.94	0.74 (0.52, 1.07)	0.1100	0.69 (0.48, 0.99)	0.0443
Huang Qin (*Rx. Scutellariae*)	3,604	11.64	2.96	0.52 (0.34, 0.79)	0.0021	0.56 (0.37, 0.86)	0.0076
Xia Ku Cao (*Spica Prunellae*)	3,564	10.68	1.55	0.80 (0.55, 1.14)	0.2155	1.00 (0.70, 1.45)	0.9824
Chuan Xiong (*Rz. Chuanxiong*)	3,442	11.31	1.33	0.34 (0.20, 0.59)	0.0001	0.37 (0.22, 0.65)	0.0005
Shan Zha (*Fr. Crataegi*)	3,138	11.50	1.26	0.67 (0.45, 1.00)	0.0520	0.79 (0.52, 1.19)	0.2540
**Multi-herb products (in Chinese)**							
Tian-Ma-Gou-Teng-Yin	18,990	10.91	7.39	0.79 (0.64, 0.98)	0.0302	0.89 (0.72, 1.09)	0.2610
Xue-Fu-Zhu-Yu-Tang	6,884	10.66	5.83	0.87 (0.67, 1.14)	0.3217	0.94 (0.72, 1.23)	0.6593
Gou-Teng-San	5,930	10.12	5.65	0.80 (0.59, 1.07)	0.1359	0.91 (0.68, 1.23)	0.5444
Jia-Wei-Xiao-Yao-San	4,549	11.30	6.86	0.48 (0.33, 0.70)	0.0002	0.60 (0.41, 0.88)	0.0086
Zhi-Gan-Cao-Tang	3,873	10.85	5.35	0.97 (0.69, 1.38)	0.8819	0.93 (0.66, 1.32)	0.6805
Ji-Sheng-Shen-Qi-Wan	3,701	12.22	6.36	1.03 (0.72, 1.46)	0.8731	0.86 (0.60, 1.23)	0.4151
Liu-Wei-Di-Huang-Wan	3,741	11.37	6.21	0.80 (0.56, 1.15)	0.2317	0.77 (0.54, 1.11)	0.1582
Zhi-Bai-Di-Huang-Wan	3,633	10.96	8.70	0.84 (0.60, 1.18)	0.3215	0.85 (0.60, 1.19)	0.3345
Bu-Yang-Huan-Wu-Tang	3,483	10.00	6.26	0.97 (0.67, 1.41)	0.8691	0.84 (0.58, 1.23)	0.3803
Qi-Ju-Di-Huang-Wan	3,356	12.10	6.49	0.86 (0.59, 1.24)	0.4052	0.74 (0.51, 1.06)	0.1039

* *Adjusted for gender, age, income, urbanization, duration of hypertension, coronary heart disease, cerebrovascular disease, diabetes mellitus, hyperlipidemia, PAOD, ACEI, ARB, α-blockers, β-blockers, CCB, diuretics, and others, i.e., hydralazine, clonidine, nitroprusside, methyldopa, minoxidil, and diazoxide*.

*ACEI, Angiotensin-Converting Enzyme Inhibitors; ARB, Angiotensin Receptor Blockers; CCB, Calcium Channel Blockers; CHM, Chinese Herbal Medicine; CI, Confidence Interval; HR, Hazard Ratio; PAOD, Peripheral Arterial Occlusive Disease*.

***Bu-Yang-Huan-Wu-Tang (BYHWT) components**: Rx. Astragali, Rx. Angelicae Sinensis, Rx. Paeoniae Rubra, Rx. Ligusticum Chuanxiong, Sm. Persicae, Flos Carthami, Pheretima; **Gou-Teng-San (GTS) components**: Ram. cum Uncis Uncariae, Rx. Ginseng, Poria, Poria Pararadicis, Rx. Ophiopogonis, Per. Citri Reticulatae, Rz. Pinelliae Preparatum, Rx. Saposhnikoviae, Flos Chrysanthemi, Gypsum Fibrosum, Rz. Zingiberis Recens, Rx. Glycyrrhizae; **Ji-Sheng-Shen-Qi-Wan (JSSQW) components**: LWDHW plus Rx. Aconiti Lateralis Preparata, Cx. Cinnamomi, Rx. Cyathulae and Sm. Plantaginis; **Jia-Wei-Xiao-Yao-San components**: Rx. Angelicae Sinensis, Rx. Paeoniae Alba, Poria, Rz. Atractylodis Macrocephalae, Rx. Bupleuri, Cx. Moutan, Fr. Gardeniae, Rx. Glycyrrhizae Preparata, Hb. Menthae Haplocalycis, Rz. Zingiberis Recens; **Liu-Wei-Di-Huang-Wan (LWDHW) components**: Rx. Rehmanniae Preparata, Fr. Corni, Rx. Dioscoreae, Rz. Alismatis, Poria, Cx. Moutan; **Qi-Ju-Di-Huang-Wan (QJDHW) components**: LWDHW plus Fr. Lycii and Flos Chrysanthemi; **Tian-Ma-Gou-Teng-Yin (TMGTY) components**: Rz. Gastrodiae, Ram. Uncariae cum Uncis, Concha Haliotidis, Fr. Gardeniae, Rx. Scutellariae, Hb. Leonuri, Rx. Cyathulae, Cx. Eucommiae, Hb. Taxilli, Caul. Polygoni Multiflori, and Poria Pararadicis; **Xue-Fu-Zhu-Yu-Tang (XFZYT) components**: Sm. Persicae, Flos Carthami, Rx. Angelicae Sinensis, Rz. Chuanxiong, Rx. Paeoniae Rubra, Rx. Cyathulae, Rx. Bupleuri, Rx. Platycodi, Fr. Aurantii, Rx. Rehmanniae, and Rx. Glycyrrhizae; **Zhi-Gan-Cao-Tang (ZGCT) components**: Radix Glycyrrhizae Preparata, Radix Ginseng, Ramulus Cinnamomi, Radix Rehmanniae, Tuber Ophiopogonis, Colla Corii Asini, Semen Cannabis, Rhizoma Zingiberis Recens, Fructus Zizyphi Jujubae; **Zhi-Bai-Di-Huang-Wan (ZBDHW) components:** LWDHW plus Rx. Anemarrhenae, Cx and Phellodendri*.

## Discussion

This report presents the first large-scale population-based retrospective cohort study providing evidence of the association between CHM use and HF risk in patients with HTN, which was conducted by analyzing claims data from the NHIRD in Taiwan. The main findings were as follows: (1) CHM use may prevent HF in hypertensive patients because it was associated with a 0.85-fold risk. This effect is related to the number of medication days, since the risk of HF in hypertensive patients who used CHM for more than 180 days/year fell to 0.65 times that in the control group. (2) Compared with controls, male hypertensive patients or those with older age, CHD, CVD or DM had a higher risk of developing HF, but CHM use could not reduce the risk of HF in male hypertensive patients or those with comorbidity. (3) The most common CHM formula prescribed was TMGTY, and the most common single herb prescribed was Dan Shen.

Of the many risk factors for HF, some can be controlled, but not others. Age and sex are two uncontrollable risk factors for HF. In general, the incidence of HF is higher in older than in younger people (18). Simply getting older could increase the risk of HF because of cardiac aging processes including fibrosis, inflammation, mechanical stiffening and diastolic dysfunction, mitochondrial dysfunction, and a growing imbalance between loss and birth of cardiomyocytes (18). Different diagnostic criteria, categorizations, and sources of patients in epidemiological research may produce different results (4). In the Framingham Heart Study, HF was greater in hypertensive women than in men (6). However, recent epidemiological research on HF reported that women have a significantly lower incidence rate of HF compared to men in all age categories except the category of age > 74 years, and HF with preserved ejection fraction is more common in women (4). The data in our study showed that hypertensive patients aged more than 65 years had a 5.3-fold higher risk of developing HF as compared with those aged < 45 years. Consistent with a previous study by Rismiati and Lee (19), our results showed that the risk of developing HF was 1.34-folds higher in male patients with HTN than in female patients with HTN. Our study results revealed that patients treated with CHM after diagnosis of HTN showed a significantly lower risk of HF in the future. In addition, better effects occurred with longer durations of CHM treatment. CHM use can reduce the risk of HF, particularly in hypertensive females and those aged more than 45 years, but not in hypertensive males. These are encouraging results, for they suggest that CHM may be beneficial for cardiac aging or reduce the increased risk of HF caused by aging, which is categorized as an uncontrollable risk factor. The HF risk was not significantly reduced by CHM in patients aged younger than 45 years and in males. The possible explanations include that hypertensive males and those diagnosed with HTN before 45 years old may have unhealthy diets or lifestyles, such as smoking or physical inactivity, obesity, or congenital heart disease (4).

The development of HF has mixed etiologies. Several etiologies, such as CHD, hypertensive heart disease, hyperlipidemia, and DM, may increase the risk of HF (20). Our results showed that patients with HTN who presented with comorbidities, including CHD, CVD, or DM, had higher risk of developing HF, but this trend was not found in those with hyperlipidemia or peripheral arterial occlusive disease. The results showed that CHM use could not reduce the risk of developing HF in hypertensive patients with concomitant diseases mentioned above. A possible reason is that the CVD continuum is a sequence of cardiovascular events, one which begins from several cardiovascular risk factors including DM, hyperlipidemia, HTN, smoking and obesity, followed by CHD or myocardial infarction, and eventually end-stage HF and death (21). Higher numbers of these risk factors are associated with greater chances of developing HF. Previous studies have shown that the prevalence of HTN is higher in low and middle-income countries than in high-income countries (3), and low socioeconomic status independently increases risk of HF (4). Our results showed that the levels of income and urbanization were not associated with higher risk of HF. The possible reasons are the increased awareness, treatment, control and compliance with medication for HTN among Taiwanese people, as well as the improvement of accessibility to medical resources in rural areas. In addition, the results of the study indicated that hypertensive patients with higher incomes or those living in rural areas had lower risk of developing HF while receiving adjuvant CHM treatment. The cause of these findings is not fully understood. One possible explanation is high-income patients who receive adjuvant CHM treatment may tend to seek and can afford more complementary and alternative medicine therapies, as well as having better health behaviors (22). Healthcare resources are often difficult to access in rural areas. Among rural residents, the utilization of CHM as adjuvant therapy with antihypertensive drugs may suggest that they tend to seek and have the ability to pursue better medical care.

According to HTN practice guidelines, the pharmacological treatment options consist of ACEI, ARB, β-blockers, CCB and diuretics (23). A previous study showed that ACEI, ARB, and thiazide diuretics appear to be most effective in preventing new-onset HF in hypertensive patients (24). Our results were not consistent with that previous study, for our study revealed that hypertensive patients who received ARB had lower risk of developing HF; those who received α-blockers, β-blockers, or CCB were not at lower risk for HF; and those who received ACEI or diuretics were at higher risk for HF. The cause of this disparity is unknown, and possible reasons include different study designs, different lifestyles, or genetic factors in other studies. In addition, patients who require treatment with β-blockers or diuretics may have other specific conditions that increase HF risk, such as DM, post myocardial infarction, atrial fibrillation, or fluid overload (23). As for ACEI, which increased HF risk in our study, one possible reason is that side effects such as dry cough may have led to patient non-adherence. Our additional results for Table 1 showed that 41.02% of CHM non-users and 40.78% of CHM users had been prescribed both ACEI and ARB during the follow-up period. We were unable to identify whether they used ACEI and ARB concomitantly or received ARB after an initial treatment with ACEI by NHIRD. However, we believe that the latter is more likely because the combination ACEI and ARB is contraindicated by Taiwan's hypertension guidelines (25). The high proportion of alternative use of ARB following initial treatment of ACEI suggested the possibility of patient non-adherence. It is especially notable that the results demonstrated that CHM use in combination with β-blockers was effective in reducing the risk of developing HF in hypertensive patients as compared with the risk in patients with no CHM use.

The top 10 most frequently prescribed formulae for treating HTN are indicated for a variety of syndromes based on TCM theory, such as TMGTY for the syndrome of internal stirring of liver wind, Xue-Fu-Zhu-Yu-Tang for the syndrome of heart blood stagnation, Gou-Teng-San for the syndrome of liver wind, JWXYS for the syndrome of liver *qi* stagnation, Zhi-Gan-Cao-Tang for the syndrome of heart *qi* deficiency, Bu-Yang-Huan-Wu-Tang for the syndrome of *qi* deficiency and blood stagnation, and Liu-Wei-Di-Huang-Wan and its derivatives for the syndrome of kidney deficiency (14). CHMs have the potential to reduce BP by single or multiple pharmacological mechanisms, such as increasing the nitric oxide (NO) level, blocking the calcium channel, inhibiting the rennin-angiotensin system (RAS), dieresis, and the central mechanism, and they also have anti-cardiac hypertrophic effects by regulating the p38/NF-κb pathway (14, 15). Our results are consistent with those of previous studies (14, 16) in that the most commonly used CHM formula was TMGTY, and the most commonly used single herb was Dan Shen. The combination of TMGTY and Dan Shen was the most common co-prescription for treating HTN (16). TMGTY is usually used to treat HTN-related symptoms in clinical practice. Multiple activities of TMGTY in treating HTN have been demonstrated, including increasing NO levels, blocking calcium channels, regulating the RAS, increasing the diuretic effect, and acting on the central vasomotor center (14, 16). TMGTY has also been reported to lower total cholesterol, prevent the stroke risk in hypertensive patients, and improve clinical symptoms and quality of life (16). Dan Shen has also been reported to have multiple activities in the cardiovascular system, including anti-HTN, anti-atherosclerosis, anti-thrombosis and cardioprotection (16). Moreover, several beneficial effects of Dan Shen in HF prevention include an anti-cardiac hypertrophic effect, reducing adriamycin-induced cardiomyopathy, and improving cardiac angiogenesis and ejection function by modulating the HIF1α/VEGFA signaling pathway after myocardial infarction (15). However, further analysis showed TMGTY and Dan Shen were unable to reduce the risk of HF. The frequently prescribed formula JWXYS and the single herbs Ge Gen, Huang Qi, Du Zhong, Huang Qin, and Chuan Xiong were significantly associated with lower risk of HF. JWXYS is rarely prescribed alone by TCM physicians for treating HTN, however, our study revealed that it was the fourth most commonly prescribed formula. JWXYS is used widely to reduce anxiety, sleep disturbance, and fatigue as well as to manage mood swings associated with climacteric syndrome (26). TCM physicians usually combined it as adjuvant formula to stabilize emotions and restore balance of the autonomic nervous system (27). Patients with such disorders might be at a higher risk of developing HTN, and this may explain the frequent prescription of JWXYS for HTN. Its modified formulation, Xiao-Yao-San (XYS), is beneficial for hypertensive patients in lowering BP, improving depression, regulating blood lipid levels, and inhibiting inflammation (28). So far, no studies have mentioned that JWXYS can prevent HF. This is a new finding that JWXYS can prevent new-onset HF in hypertensive patients. CHM contains numerous active components or phytochemicals that may have multifaceted mechanisms of cardioprotective effects, including antioxidant, vasorelaxant, anti-inflammatory, anti-proliferative, or diuretic effects (29). Five frequently used single herbs, namely, Ge Gen, Huang Qi, Du Zhong, Huang Qin, and Chuan Xiong, were found to reduce the risk of HF in hypertensive patients. The possible mechanisms involved in preventing HF at least include inhibition of angiotensin converting enzyme or angiotensin II receptor (29–32).

The strengths of this study include the comprehensiveness of the nationwide database, which eliminated the possibility of recall bias in questionnaire assessments and ensured that CHM was used before HF occurred. In addition, the study used a multivariate adjusted hazard ratio with a period of 16 years follow-up to confidently estimate the effects of CHM therapy on associations between HTN and the risk of HF over a long latency period. Despite these strengths, several limitations should be noted when interpreting the results of the present study. First, it was difficult to obtain the results of BP levels, body weight measurements, body mass index, medical compliance, lifestyle, and other clinical characteristics, including smoking, alcohol consumption and education level, for individual patients in detail from the NHIRD. The failure to adjust for putative risk factors may have resulted in biased estimates of risks of HF in our sample. Second, other preparations of Chinese herbal remedies, health foods containing natural herbs, folk medicine, and direct purchases from TCM herbal pharmacies are not reimbursed by NHI and thus were not analyzed in this study. However, the high healthcare insurance coverage and low prices of government-approved CHM have led to a reduction in herbal folk medicine use. This missing data could possibly have resulted in underestimation of the cumulative frequencies and may have weakened the effect of the specified CHM, but the effect, if any, should be negligible.

## Conclusion

This 16-year follow-up cohort study found that the use of CHM during the treatment of HTN was associated with a 15% lower risk of developing HF compared with the risk among CHM non-users, and the effect increased to a 35% lower risk with a duration of CHM treatment > 180 days. Our study results suggest that the use of CHM as an adjuvant therapy in hypertensive patients may prevent subsequent HF. Further long-term prospective studies or randomized, placebo-controlled clinical trials are needed to validate these findings.

## Data Availability Statement

The original contributions presented in the study are included in the article/supplementary material, further inquiries can be directed to the corresponding author.

## Ethics Statement

The studies involving human participants were reviewed and approved by China Medical University Hospital. Written informed consent for participation was not required for this study in accordance with the national legislation and the institutional requirements.

## Author Contributions

M-YT and C-TL designed the study and wrote the manuscript. CH, and I-LH acquired the data. K-CH analyzed the data. M-YT and Y-HC revised the manuscript. All authors read and approved the final manuscript.

## Funding

This study was supported in part by the Taiwan Ministry of Health and Welfare Clinical Trial Center (MOHW 110-TDU-B-212-124004), and China Medical University Hospital (DMR-111-105). We are grateful to the Health Data Science Center, China Medical University Hospital, for providing administrative, technical, and funding support. The funders had no role in the study design, data collection and analysis, the decision to publish, or preparation of the manuscript. No additional external funding was received for this study.

## Conflict of Interest

The authors declare that the research was conducted in the absence of any commercial or financial relationships that could be construed as a potential conflict of interest.

## Publisher's Note

All claims expressed in this article are solely those of the authors and do not necessarily represent those of their affiliated organizations, or those of the publisher, the editors and the reviewers. Any product that may be evaluated in this article, or claim that may be made by its manufacturer, is not guaranteed or endorsed by the publisher.
